# Papillary Thyroid Carcinoma: Differential Diagnosis and Prognostic Values of Its Different Variants: Review of the Literature

**DOI:** 10.5402/2011/915925

**Published:** 2011-12-06

**Authors:** Rogelio Gonzalez-Gonzalez, Ronell Bologna-Molina, Ramón Gil Carreon-Burciaga, Marcelo Gómezpalacio-Gastelum, Nelly Molina-Frechero, Sirced Salazar-Rodríguez

**Affiliations:** ^1^Universidad Autónoma Metropolitana, México City, Mexico; ^2^School of Dentistry, Research Department, Universidad Juárez del Estado de Durango (UJED), Durango, Mexico; ^3^Health Care Department, Universidad Autónoma Metropolitana, Xochimilco, Mexico City, Mexico; ^4^Instituto Nacional de Oncología y Radiobiología (INOR), La Habana 10400, Cuba

## Abstract

Papillary thyroid carcinoma is the most common thyroid malignancy, and has an excellent prognosis, even with cervical lymph node metastasis; however, histological variants are considered relevant, which may be associated with familial adenomatous polyposis and tumor aggressiveness. Histological features, such as vascular and/or lymphatic invasion, angiogenesis, multifocality, high cellular proliferation rate, neoplastic cell dissemination, and the histological varieties, are indicative of poor prognosis, together with associated clinical factors: age, sex, and tumor size.

## 1. Introduction

Papillary thyroid carcinoma (PTC) represents 1% of all malignancies [1] and represents 70–80% of all thyroid cancers. Several factors, are associated with the development of this neoplasm, including genetic alterations, growth factors and radiation [2]. The prognosis of this tumor is strongly associated with various clinical variables as follows: age, tumor size, and histological parameters such as extracapsular extension, extrathyroidal extension, lymph node invasion, distant metastasis and histological variants.

Clinical and histological features are used to classify these carcinomas in different clinical stages, which influences the treatment plan and prognosis for survival of patients with PTC. *RET/PTC* is an important oncogene in the initiation events in the pathogenesis of cancers and is rearranged, particularly in patients who have been exposed to radiation. The activation of the *BRAF* gene accounts for approximately 45% of sporadic mutations that result from increased *BRAF* kinase activity. Approximately 80% of all mutations have transversion events, that is, the transversion from thymine to adenine at nucleotide 1799 [3].

The purpose of this study was to conduct a review of various publications with respect to tumor biology, risk factors associated with the development of PTC, histological and clinical features, and treatment of PTC.

## 2. Review of the Clinical Features and Metastasis of Papillary Thyroid Carcinoma

**Figure 1:**
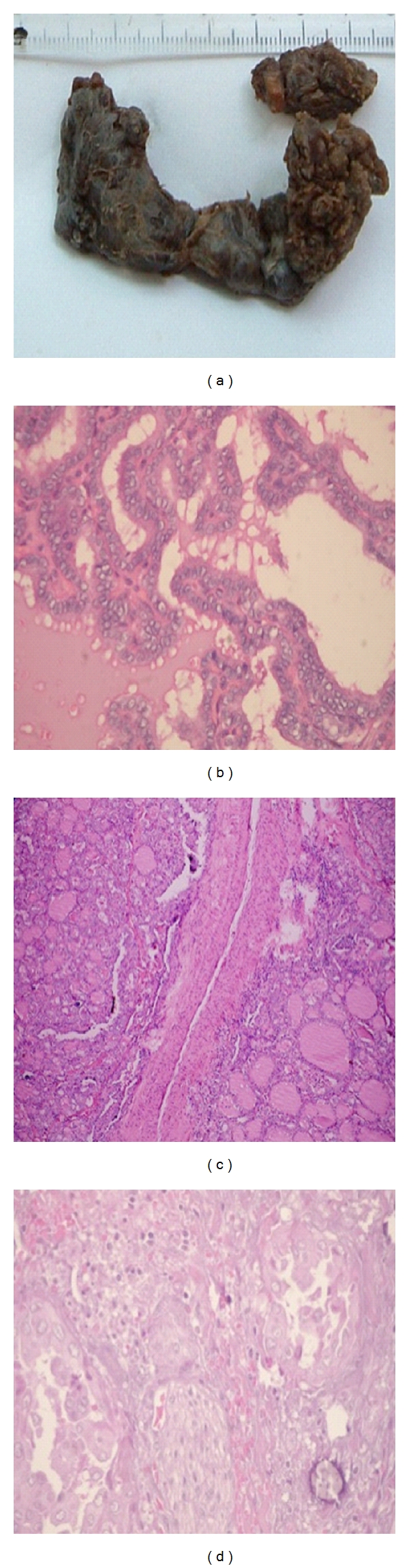
(a) Total thyroidectomy in PTC with extracapsular invasion, (b) PTC classical/conventional [20x], (c) vascular invasion in PTC classical/conventional (4x), and (d) nerve invasion in PTC [20x].

Different systems of classification (AMES, AGES, MACIS, EORTC) were used to predict the risks of PTC. These systems classify different features such as the following: age, gender, size, extension, and distant metastases. TNM classification evaluates the prognosis of the disease associated with nodal status [4]. TNM is a system that accurately predicts life expectancy [5]. Most metastases are often found in lymph nodes, may affect the clinical course of the injury, may be indicative of distant metastases, and have significant effects on the course of the disease [6]. Lymphatic metastasis may be associated with features such as multifocality, patient age, local recurrence, and distant metastases regardless of tumor size and extrathyroidal extension, especially those occurring in the central region of the neck [4, 7, 8]. Studies by Wada et al. [6] have indicated that the presence of lymph node metastases occur more frequently and are associated with a higher risk of recurrence in younger patients (<45) than in older (≥45) patients. However, recurrences of PTC are more frequent in older patients than younger patients, independent of metastasis. Therefore, the increase in the number of lymph node metastases may increase the risk for a worse prognosis. Results of clinical studies indicate that older patients who have lymph node metastases have a worse prognosis than younger patients. Lymphatic metastasis at the cervical level does not seem to affect patient survival, although the risk of local or regional recurrence is increased [9]. Ultrasonography is a method of choice for the detection and diagnosis of cervical lymph node metastases in patients with PTC. Ultrasonography is used preoperatively and displays a sensitivity of 51–62% and a specificity of 79–98% [10]. Detection of occult metastases can be treated with prophylactic radioiodine to avoid radical procedures. However, treatment of evident nodal disease necessitates radical lymphadenectomy. Distant metastasis of PTC are rare and usually occur in advanced stages of the disease, especially in lung, bone, lymph nodes of the chest, pancreas, and breast [11]. Some variants of PTC are associated with poor prognosis and are characterized by tall cells as well as columnar and insular patterns [12]. The management of PTC is variable and depends on the characteristics of each tumor; many cases have been subjected to extensive surgery, radioactive iodine ablation or external beam radiotherapy. Radioactive adjuvant therapy (RAI) is used to treat differentiated thyroid disease, eradicate microscopic diseases, and detect early recurrences due to increased measurement sensitivity of this material [5]. In PTC patients, the effectiveness of RAI to decrease the risk of relapse of the disease is reduced by up to 54%. However, RAI may prolong the life expectancy in apparently disease-free patients following surgery [5]. Age is considered a major risk factor, as it has been shown that patients over 45 years displayed an increased risk of death by 5.4- to 6.05-fold [5, 13]. Therefore, total thyroidectomy favors increased life expectancy compared to lobectomy. Several authors mention that treatment with total thyroidectomy is associated with several complications. However, repeated surgeries following total thyroidectomy were reported in less than 5% [14] (Figure 1).

## 3. Review of Variants of Papillary Thyroid Carcinoma

Many morphological variants of PTC have been described, and their different behaviors have been characterized [15].

## 4. Diffuse Sclerosing Variant (DSVPC)

**Figure 2:**
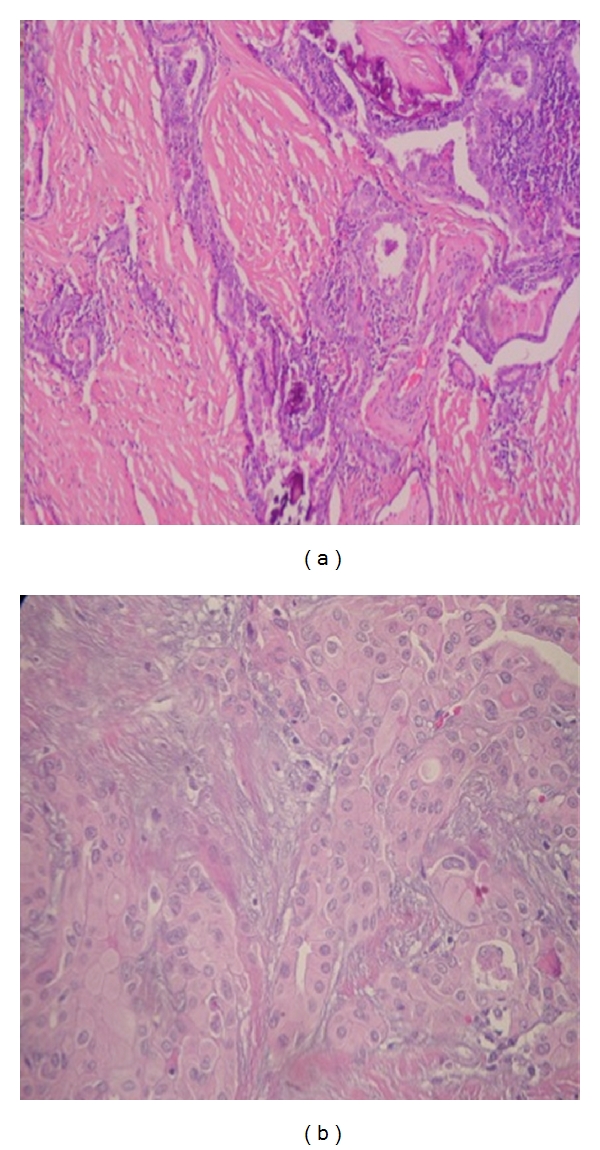
(a) DSVPC. Tumour replaces the lobe of gland, accompanied by dense fibrosis (20x), (b) squamous metaplasia [20x].

DSVPC is a rare variant of papillary thyroid carcinoma detected in 1.8% of PTC cases evaluated in large studies. DSVPC is commonly seen in young, this neoplasm ranging from 19.5 to 34.7, years and has a higher incidence of cervical lymph node metastases [16, 17]. Several authors have suggested that the prognosis may be unfavorable and, therefore, requires aggressive treatment [18]. The clinical features may include palpable thyroid nodules associated with unilateral and ipsilateral or bilateral cervical metastases. The multifocality, bilaterality and extra-thyroid extension are observed more frequently in this carcinoma compared with the conventional variant. [19]. Ultrasonographic studies of this variant, are frequently observed, dispersed calcification in both lobes and diffuse and hypoechoic nodules that are diffuse and ill defined. In addition, these studies indicate that most tumors grow to approximately one centimeter in diameter. Radiography detects microcalcifications that may not be visible on ultrasounds and may be related to psammoma bodies. Therefore, these radiological features may predict the potential extent of thyroid cancer. Histology is used to characterize dense fibrosis, extensive squamous metaplasia, lymphoid infiltration in the presence of germinal centers, and psammoma body formation with areas of conventional PTC [16] (Figure 2). Immunohistochemical studies are used to evaluate thyroglobulin, TTF-1, HBME1, and galectin-3 in PTC [20]. The aggressiveness of this type of PTC may be associated with extrathyroidal extension, cervical lymph node metastasis, and distant metastasis associated with advanced clinical stages. [21]. However, these characteristics have been controlled to improve prognosis using aggressive surgical treatment, RAI, and external radiotherapy, which has worked to reduce locoregional relapse in patients with locally advanced disease [22]. Falvo et al. [23] in a study of 83 patient with the diffuse sclerosing variant of PTC and 168 patients with classical PTC illustrated that the incidence of laterocervical lymph node pathology at diagnosis was significantly higher for the diffuse sclerosing variant. Studies made by Lam and Lo [17] concluded that complete resection did not result in later recurrence in any of the patients; therefore, cosmetic and complication-free surgery should be considered. Additionally, the rates of recurrence and distant metastases were increased compared with those for classical PTC patients.

Conventional PTC rearrangements of *RET/PTC* are associated with high prevalence rates in children and young adults; the age of presentation is significantly lower compared to other types of PTC. Therefore, it is not surprising that the presence of *RET/PTC* is associated with initial genetic events such as the DSVPTC and that these tumors are susceptible to RET therapy [24, 25].

## 5. Tall Cell Variant (TCVPC)

**Figure 3:**
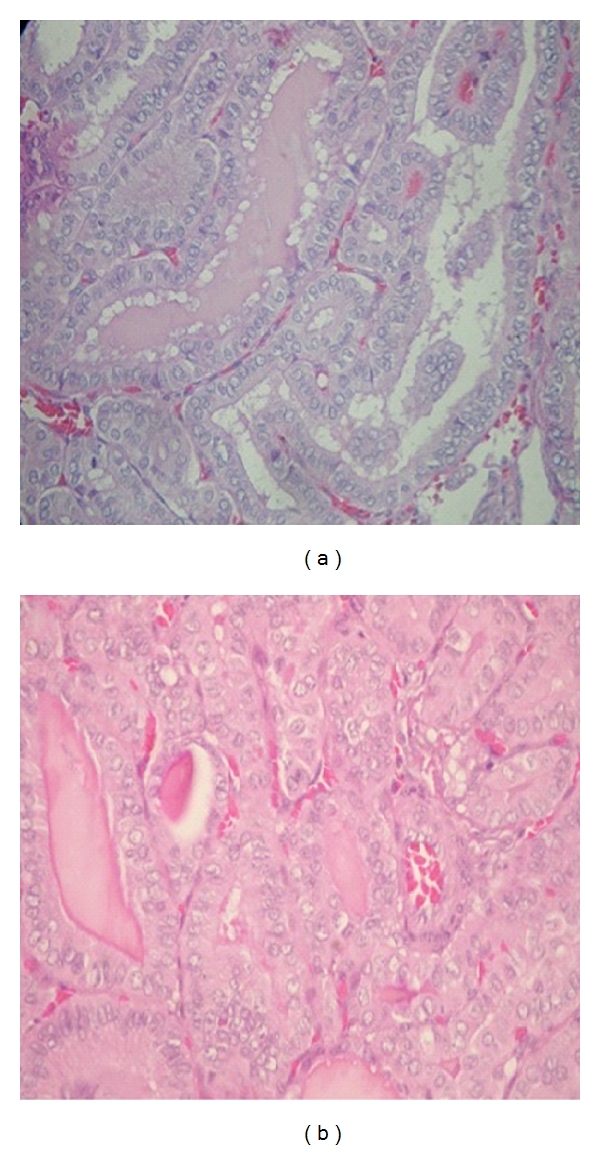
(a) TCVPC typically appearance [20x]; (a,b) TCVPC shows characteristic cytologic features, including elongate cells, intracytoplasmic borders, and eosinophilic cytoplasm [20x].

The TCVPC was first described in 1976 by Hawk and Hazard as an aggressive variant of PTC [26]. TCVPC is defined as a tall-cell papillary carcinoma containing cells that are at least two times higher in height than width with nuclei that are oriented to the basement membrane. To consider TCVPC, there must be 30%–50% tall cells in the tumor [27]. The TCVPC represent 3.8–10.4% of PTC, and histology indicates that TCVPC is a poor prognostic factor when considered in isolation [28]. Factors such as clinical stage, grade, and age (over 50 years, range 34–72 years) are related to prognosis [29]. A meta-analysis study conducted by Jalisi et al. [28] has indicated that the prognosis for TCVPC is poor because these tumors represent high local recurrence, lymph node metastasis, and mortality. These results suggest the necessity for aggressive treatment with at least total thyroidectomy. The ultrasound results reveal microlobulated hypoechoic nodules with microcalcifications that are associated with extrathyroidal extension and cervical lymph metastasis [29]. TCVPC occupies at least 30–50% of the tumor. The cytoplasm of the cells is abundant and eosinophilic. Oncocytic differentiation, syncytial cell growth, and capsular and vascular invasion are observed [27] (Figure 3). In addition, differentiation or squamous metaplasia is caused by persistent thyroglossal duct- or branchial pouch-derived structures. These features of squamous cells lead to the transformation of squamous cell carcinoma such as undifferentiated squamous cell carcinomas, especially the spindle cell type [30], which indicate poor prognosis and survival. The receptor tyrosine kinase c-Met interacts with the hepatoctic growth factor (HGF). Overexpression of c-Met induces cell motility and invasion. In addition, c-Met overexpression promotes the aggressive and invasive behavior of cells in thyroid carcinoma [31–33]. In a study of the mitogenicity of *RET/PTC3* by Basolo et al. [34], the CTV *RET/PTC3* oncogene was detected in 14 of 39 (35.8%) cases studied. This result may indicate a more aggressive oncogenic potential, which may explain the behavior of TCVPC and may suggest discarding the idea that the aggressiveness of TCVPC is correlated to the clinical features.

The aggressive behavior of the tall cell variant may also be related to the higher prevalence of activating point mutations of BRAF in the tall cell variant than in classical PTC [35]. Indeed, papillary cancers of any subtype that have BRAF mutations have a higher frequency of extraglandular extension and nodal metastases. The incidence of the tall cell variant is accentuated by the fact that it is overrepresented in thyroid carcinomas that are RAI found that 20% of fluorodeoxyglucose positron-emission tomogram (FDGPET) positive/RAI refractory [36].

## 6. Solid Variant (SVPC)

The SVPC should be considered when representing more than 50% of the tumor mass because different variants of PTC may occur as focal solid patterns. This variant occurs frequently in children, and over 30% of the reported cases are associated with the Chernobyl nuclear accident [37]. Studies that were conducted in Japan indicate that the SVPC is aggressive and has a poor prognosis. However, these findings have not been confirmed in Europe and North America. These tumors should not be grouped with other tumors that are more aggressive but should be characterized as poorly differentiated carcinomas with insular patterns [38].

In a study made by Nikiforov et al. [39] in 20 primary cases of SVPC, they found that this variant is associated with a slightly higher frequency of distant metastases and less favorable prognosis than classical PTC.

Silver et al. [20] recommend in aggressive variants of PTC including the SVPC that The surgeon must be prepared to perform a total thyroidectomy, central compartment neck dissection, additional lymphadenectomy and/or resection of invaded surrounding structures, and search for distant metastasis.

## 7. Papillary Thyroid Microcarcinoma (PTMC)

PTMC is defined as carcinoma of the thyroid gland and is undetectable during the preoperative clinical examination. Therefore, PTMC is diagnosed as a benign lesion. Removal of the thyroid gland can reveal the presence of a tumor, which is less than 10 mm at its widest diameter [40]. The PTMC can be indirectly detected because the diagnosis is indicated by the presence of cervical lymph node metastases. [37]. This type of carcinoma has also been given the name of occult papillary carcinoma, which describes PTC foci less than 1–1.5 cm [41]. Aspiration biopsy that is guided by ultrasound is relatively sensitive with 60–90% specificity, a positive predictive value of 100%, a negative predictive value of 80%, and a precision rate of up to 85% for the diagnosis of thyroid cancer. PTMC can be diagnosed in lesions that are greater than 3 mm. Ultrasound-guided biopsy can be used for patients with suspicious cervical lymph nodes [41]. If PTMC appears benign, conservative surgery is the treatment of choice. However, the patient should be kept under surveillance because up to 11% of patients have metastases [42]. The PMC variant is the most common form of PTC that is identified at autopsy and in nonthyroid pathologies. Once detected and treated, most of these tumors do not metastasize, and in some studies, they have no effects on mortality. Previous studies have reported metastasis to the lymph nodes in 3–15% of cases with a mortality rate of 1% [43]. Studies by Bologna-Molina et al. [44] on syndecan protein-1 (SDC-1) demonstrated that noninvading PTMC cells expressed lower levels of SDC-1 compared to extracapsular invading PTMC cells. These results show that the overexpression of SDC-1 in PTMC may be related to tumor progression. The tumor progression may be associated with multifocality to promote metastasis. A study by Dunki-Jacobs et al. [45] has shown that multifocality is often associated with cervical metastasis. A study by Hay et al. [13] found that, in 900 cases, 23% of PTMC was associated with the presence of metastasis and that only one patient had evidence of distant metastasis. The findings of this study are similar to those by Dunki-Jacobs et al. [45], and Bologna-Molina et al. [44] indicate that multifocality is a major factor for tumor progression. PTMCs are generally well-differentiated neoplasms, which show characteristic architecture as well as cytological and immunohistochemical features. In addition, the nuclei have a frosted glass appearance and have nuclear grooves that invaginate into the cytoplasm. Other notable features are the presence of desmoplasia and the proximity of the tumor near the capsule [41]. Immunohistochemical studies reveal the conventional pattern of PTC including the injury-positive markers TTF-1 and thyroglobulin, Galectin-3, and HBME-1. The arrangements of RET/PTC1, which have been linked to aggressive biological behaviors, indicate molecular changes that are observed in the early stages of carcinogenesis but are not required for tumor progression [41]. The PTMC may include *BRAF* mutations that are associated with extrathyroidal extension and cervical lymph node metastases. Therefore, *BRAF* mutations may be useful to predict the recurrence of these neoplasms [46].

## 8. Columnar Cell Variant (CCVPC)

The CCVPC is a rare subtype of PTC, and many cases of CCVPC are aggressive. Encapsulated CCVPC occur in young or female patients, and extracapsulated CCVPC occur in older or male patients. CCVPC is described as an aggressive form of PTC and usually correlates with a poor prognosis, which may even lead to death [47–49]. However, encapsulated CCVPC (ECCVPC) is a rare variant of CCVPC representing between 0.15 and 0.2% of all PTCs [50]. In general, the CCVPC is characterized by extrathyroidal extension with cervical metastasis and distant metastases using diagnostic histopathology. This variant may be identical to TCVPC with the exception that TCVPC is characterized by papillae that are delineated by a single layer of tall cells with an abundant acidophilic cytoplasm and the presence of granules that give an oncocytic appearance. CCVPC induces stratified prominent, clear cytoplasm that is reminiscent of subnuclear vacuolated cells, resembling secretory endometrium [21]. The neoplastic cells are positive for thyroglobulin and TTF-1. In CCVPC, V600E mutations in the *BRAF* gene are detected and may be associated with increased cyclin D1, Ki-67 proliferation, and nuclear expression of estrogen and progesterone [51].

## 9. Clear Cell Variant (CLCVPC)

CLCVPC is defined by the World Health Organization (WHO) [52] as a neoplasm that is predominantly composed of clear cells and papillary or follicular growth and mainly affects women between the sixth and seventh decade of life [53]. Histologically, CLCVPC is characterized by clear cytoplasm, which is probably due to TSH overstimulation. The nuclear features are similar to conventional PTC. Immunohistochemical studies reveal that CLCVPC is characterized by TTF-1, thyroglobulin, HMBE, and Galectin-1 [15].

## 10. Cribriform-Morular Variant (C-MVPC)

C-MVPC of PTC is a rare morphologic entity. It was first described by Harach et al. [54] in association with familiar adenomatous polyposis (FAP) as a distinctive tumor. C-MVPC of PTC is common in young females usually less than 30 years of age. The lesions are encapsulated or well circumscribed. Although sporadic forms of C-MVPC usually appear as isolated tumors, the cases that are associated with FAP are often multifocal because different somatic mutations are added to the germline mutations [55, 56]. They display the characteristic histologic pattern of cribriforming akin to that seen in breast cancer with morules [57]. This tumor type is characterized by a prominent cribriform pattern of growth with interspersed squamoid islands (morules) that frequently harbor nuclei filled with lightly eosinophilic, homogeneous inclusions, closely packed follicles, papillae, and trabecular. Characteristically, the luminal spaces are devoid of colloids. The tumor cells are columnar or cuboidal, and the nuclei are often chromatin rich. The nuclear grooves, pale or clear nuclei, and intranuclear cytoplasmic inclusions are often observed focally. Some tumor cells can be plump and spindle shaped, forming fascicles or whorls. The neoplasia is often circumscribed or even encapsulated, with or without capsular and/or vascular invasion [58]. The presence of squamous morula has been immunohistochemically analyzed by Hirokawa et al. [59] to differentiate them from squamous metaplasia, which reveals the following results: squamous metaplastic cells are immunopositive for low- and high-molecular-weight cytokeratin and show intense cell membrane staining for beta-catenin but not Bcl-2. Conversely, the morular cells are positive with Bcl-2 and negative or weakly positive for cytokeratin and beta-catenin. S-100 protein-positive dendritic cells are observed in the metaplastic nests but not in morules. Finally, Hirokawa et al. [59] concluded that morules are associated with aberrant nuclear and cytoplasmic localization of beta-catenin and are not an early form of squamous metaplasia. In 1994, Harach et al. [54] first characterized thyroid carcinoma developing in patients with FAP as a distinct follicular cell tumor in view of its histologic differences from papillary and follicular carcinoma. Genetic studies by Cameselle-Teijeiro et al. [60], have identified mutations in the *BRAF* gene and have demonstrated the association of *RET/PTC* and *PAX8-PPAR* with familial polypoid adenomatosis. The association of C-MVPC with FAP has been previously reported. However, Shubadha and Bagwan [61] explained that the potential absence of polyps during colonoscopy and germline mutations in the adenomatous polyposis coli (APC) gene provide evidence that the tumor is a sporadic counterpart of FAP-associated thyroid carcinoma. C-MVPC induces a behavior that is similar to conventional PTC, including the frequent occurrence of cervical metastases.

## 11. Oncocytic Variant (OCVPC)

Oncocytic thyroid neoplasms are characterized by aberrant levels of mitochondria. These tumors can occur anywhere in the thyroid [62]. DeLellis and Williams [52] describe the OCVPC with mahogany coffee-like cells in the papillary or follicular architecture. OCVPC-mediated biological behavior is variable based on the series reported and the cases studied. Several studies have not indicated differences between OCVPC and conventional PTC, whereas others have suggested that OCVPC is more aggressive than conventional PTC [63]. OCVPC may be confused with other oncocytic tumors. The histologic features of the OCVPC include the proliferation of sheets of large, cohesive cells with abundant eosinophilic cytoplasm that may be unencapsulated, ill-defined boundaries, extensive infiltration of the surrounding thyroid parenchyma, follicles with abortive papillae, or papillary growth. The vast majority of these tumors have clear nuclei. “Orphan Annie” nuclei have somewhat irregular nuclear membranes with prominent peripheral margination of chromatin and inconspicuous or absent nucleoli. No psammoma bodies or areas of calcification have been observed. Different degrees of stromal fibrosis have been noted throughout the lesions [64]. The most important consequence is papillary Hurthle's cell carcinoma [65]. The nuclear features of Hurthle's cell carcinoma differ from those of oncocytic papillary carcinoma in that the former are characterized by round and vesicular nuclei and prominent centrally placed nucleoli. Focal hyperchromasia, binucleation, and marked nuclear atypia are common features of these cells [66]. Other diagnostic considerations in the histological differential diagnosis of these tumors include the TCVPC [66] and the oncocytic variant of medullary carcinoma of the thyroid. In the tall-cell variant of papillary carcinoma, the cells are also characterized by ample acidophilic cytoplasm. However, the typical granularity that results from the packing of mitochondria, which is a hallmark of oncocytic cells, is lacking, and the cytoplasm appears glassy, probably as a consequence of the accumulation of intermediate filaments [67]. Berho and Suster [64] have stated that OCVPC is variable, has a low grade, and induces behavior similar to that presented by conventional PTC.

## 12. Follicular Variant (FVPC)

FVPC is the third most common type of PTC, following conventional papillary thyroid carcinoma (CPTC) and papillary microcarcinoma [68]. Patients with FVPC often present with a larger tumor size and at a younger age than patients with CPTC [69]. FVPC also shows less calcification, psammoma bodies, and bone formation compared to CPTC. Furthermore, FVPC has more favorable clinicopathological features and a lower tumor risk group profile than CPTC. On the other hand, the long-term outcome of FVPC patients is similar to that of CPTC patients [70]. Fine-needle aspiration (FNA) and frozen sectioning (FS) are techniques that are routinely used to evaluate thyroid nodules. In the case of classic PTC, distinct nuclear features have been described that make definitive diagnosis with FNA possible [71]. In cases of FVPC, intraoperative FS and cytologic study are used to identify the specific nuclear characteristics that define PTC. This feature is distinct from FS of follicular neoplasms, which indicates that the follicular architecture is identical in adenomas and carcinomas. Because the nuclear features may be obscured by freezing artifacts in FS, intraoperative cytologic study is currently recommended when a diagnosis of FVPC is in question [72]. Kesmodel et al. [73] have shown that treatment with total thyroidectomy is not necessary in all patients, thereby decreasing the number of complete thyroidectomies. However, there are several arguments to support total thyroidectomy in this group of patients. Studies have shown that recurrence rates are lower after total thyroidectomy because PTC may be characterized as a multinodular and bilobar disease [74]. However, the disease may not be adequately managed by thyroid lobectomy alone. In addition, total thyroidectomy facilitates the use of postoperative radioiodine to ablate residual disease and enables endocrinologists to monitor patients for recurrence based on thyroglobulin levels [74]. FVPC has created continuous diagnostic controversy among pathologists. FVPC is the most difficult to differentiate from other benign thyroid and malignant thyroid lesions clinically and pathologically. The concordance rate for the diagnosis of FVPC between endocrine pathologists is less than 40% [75]. It is likely that the recent increase in the incidence of thyroid cancer is related to the mislabeling of some cases of the benign mimics of PTC as FVPC [76]. *BRAF* is a serine/threonine kinase and a member of a family of RAF genes that are an integral part of one of the major pathways controlling cellular growth and differentiation. *BRAF* is a commonly studied gene in thyroid cancer in recent years. *BRAF* functions primarily as a signal transducer between other proteins and the *MAPK/ERK* kinase (MEK) via phosphorylation of the *BRAF* gene [77].

## 13. Conclusions

PTC is a common malignant neoplasm with excellent clinical behavior, despite the presence of cervical lymph node metastases; however, there are histologic variants, which can modify the course of these neoplasms. Therefore, we consider in this paper these variants of particular importance in histopathological diagnosis because of biological behavior and clinical characteristics of each of these variants.

The purpose of this study was reviewing the different variants of PTC with different histopathological patterns compared with those with classical PTC. Different variants or histological patterns may coexist in the a same tumor.

The presence of an aggressive variant of papillary carcinoma should alert the surgeon that he is dealing with a potentially aggressive tumor. Clinical treatment decisions should be based on the stage of the disease, influenced by the knowledge that the aggressive variants tend to be associated with higher risk factors [20].
